# Effects of Removal Conditions on Mercury Amount Remaining in the Oral Cavity and inside Drainage System after Removing Dental Amalgams

**DOI:** 10.3390/ijerph182413135

**Published:** 2021-12-13

**Authors:** Yoshiki Ishida, Harumi Aoki, Taira Miyasaka, Yusuke Aoyagi, Daisuke Miura, Akikazu Shinya

**Affiliations:** 1 Department of Dental Materials Science, School of Life Dentistry at Tokyo, The Nippon Dental University, Tokyo 102-8159, Japan; haruaoki@tky.ndu.ac.jp (H.A.); miyasaka@tky.ndu.ac.jp (T.M.); yusuke-a@tky.ndu.ac.jp (Y.A.); daisuke@tky.ndu.ac.jp (D.M.); akishi@tky.ndu.ac.jp (A.S.); 2 Department of Prosthetic Dentistry and Biomaterials Science, Institute of Dentistry, University of Turku, 20520 Turku, Finland

**Keywords:** dental amalgam, mercury, Minamata Convention on Mercury, dentistry, mercury pollution

## Abstract

Mercury is produced and drained into the environment by removing dental amalgams, which may cause mercury pollution. This study aimed to clarify the mercury amount remaining in the oral cavity and inside the drain system after removal. The effects of the removal conditions and differences in drainage systems were also investigated. Dental amalgams filled in the tooth and placed in a phantom head were removed using an air turbine under several conditions (two removal methods, absence of cooling water, and intraoral suction). Then, the oral cavity was rinsed with 100 mL of water (oral rinse water), and 500 mL of water was suctioned to wash the inside of the drainage system (system rinse water). Both water samples were collected in two ways (amalgam separator and gas-liquid separator), and their mercury amounts were measured. It was found that the amount of mercury left in the oral cavity and drainage system after dental amalgams removal could be reduced when the amalgams were removed by being cut into fragments as well as using cooling water and intraoral suction. In addition, using amalgam separators can significantly reduce the amount of mercury in the discharge water and prevent the draining of mercury into the environment.

## 1. Introduction

Dental amalgams were used in daily clinical practice because of their ease of use, long-term performance, and high economic efficiency for a long time [1,2]. However, some dental amalgam components have been reported to cause hypersensitivity reactions [2,3,4,5]. In addition, dental amalgams lead to the development of amalgam tattoos that appear as dark gray-to-blue, flat macules in the gingiva [6]. Kallus and Mjör found oral lichenoid reactions in the oral mucous membrane adjacent to dental amalgams [7], and that oral lichenoid reactions might correlate with allergic sensitivity caused by mercury and mercury compounds [8]. Some studies have shown that lesions can be improved by removing dental amalgams [9,10,11,12].

Moreover, the demand for esthetic restorations is increasing, which has led to the popularity of using esthetic materials, such as composite resins and ceramics, for tooth restorations. Kusumawardani et al. assessed patient satisfaction with composite resin and amalgam restorations [13]. Consequently, most patients preferred composite resin restoration due to the aesthetics. Dental amalgams are metal materials; therefore, the color significantly differs from that of natural teeth. Considering the aesthetics, removing dental amalgam restorations and replacing them with other esthetic materials are necessary [8].

Typical components of dental amalgam are mercury, silver, copper, tin, and zinc [14,15,16]. Mercury, which is the main component (approximately 50% by weight), is a major concern. Mercury, one of the heavy metals, is known for its toxicity, and there are some well-known public health disasters that occurred due to mercury pollution such as Minamata disease occurring in Kumamoto Prefecture Japan [17,18,19,20] and Iraq poison grain disaster in Iraq [17,19,21,22,23].

On 10 October 2013, the “Minamata Convention on Mercury” was signed by 128 countries to deal with mercury pollution internationally [24,25]. Moreover, according to Annex A, part II of the Convention, the use of mercury in dental amalgam must be reduced. In fact, the use of dental amalgam is limited in some countries [1,2,14,26,27]. However, there would be many chances to remove dental amalgams because they have been used for almost two centuries [28].

It is known that the removal of dental amalgams produces mercury [1,2,14,16,27,28,29,30,31,32], and the mercury would be released into the discharged water, which could then be transferred into the environment through the drain. Once mercury is released into the environment, its reaction becomes complicated due to chemical, physical, and biological factors [24]. Moreover, mercury may change to methylmercury under specific conditions, and methylmercury is more toxic and is easily absorbed by organisms. In addition, biological concentration might occur, which would be more harmful to human health [24]. A previous study investigated the amount of mercury in the discharged water during dental amalgam removal [29], and they found a high level of mercury in the discharge water. Thus, it is clear that the discharge water during the amalgam removal was contaminated with mercury. The water including mercury, is drained through the drain system, so not only the oral cavity but the inside drain system might be contaminated. However, the amount of mercury remaining in the oral and drain system after dental amalgam removal has not been investigated yet.

On the other hand, previous studies investigated the amount of mercury vapor during removing dental amalgam fillings with various conditions [28,31]. Consequently, it was concluded that the removal conditions, such as cooling water spray and intraoral suction, had affected the results. In addition, amalgam separators are installed in dental offices to collect and prevent releasing mercury into the environment in some countries [16,27]. Therefore, this study aimed to clarify the amount of mercury in the oral cavity and drainage system after removing dental amalgams. In addition, the effects of the removal methods, presence of suction, and difference in drainage systems on the results were also investigated.

## 2. Materials and Methods

### 2.1. Tooth Preparation

**Figure 1:**
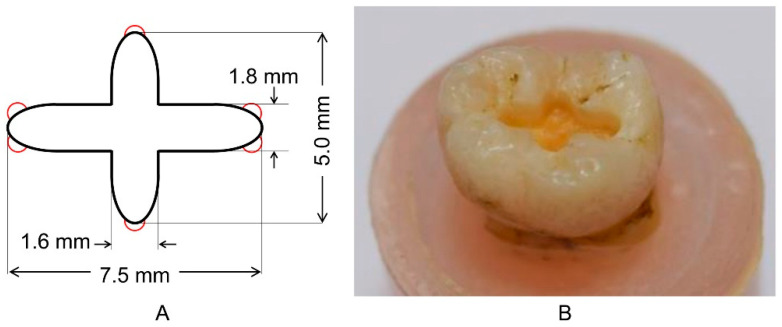
Cavity for filling dental amalgams. (**A**) dimension of the cavity. Red circles indicate dentin undercuts, (**B**) cavity prepared in the occlusal plane of a molar.

For filling dental amalgams, 80 caries- and restoration-free extracted molars were obtained according to protocols approved by the research ethics committee at the Nippon Dental University (NDU-T2013-17). To standardize the tooth position in parallel, the plane was composed of three cusps: the mesial buccal and lingual, and distal buccal. In the base plane of the mold, the teeth were placed in acrylic-cylindrical molds (15 mm of inner diameter) using self-curing resin (Ostron II, GC, Tokyo, Japan), and the height of the tooth, the distance between the occlusal plane and the base plane of the mold, was set to 25 mm. A 2-mm depth of cross-shaped cavity (Figure 1) was prepared in the center of the tooth using a cavity duplication machine (prototype device, Tokyo Giken, Tokyo, Japan) [33,34,35,36] with a diamond point (123, Horico Dental, Berlin, Germany). Then, dentin undercuts were prepared using an inverted cone bur (653-002, Tokyo Dental Industrial, Tokyo, Japan). A capsule of dental amalgam (Logic Plus, Shofu, Kyoto, Japan) was placed in an amalgam mixer (Spherical-D, Shofu, Kyoto, Japan) and mixed according to the manufacturer’s instructions. The cavity prepared at the center of the tooth was filled with dental amalgam, and the teeth with dental amalgam were stored in deionized water at 37 °C for seven days.

### 2.2. Instruments Used in This Stud

**Figure 2:**
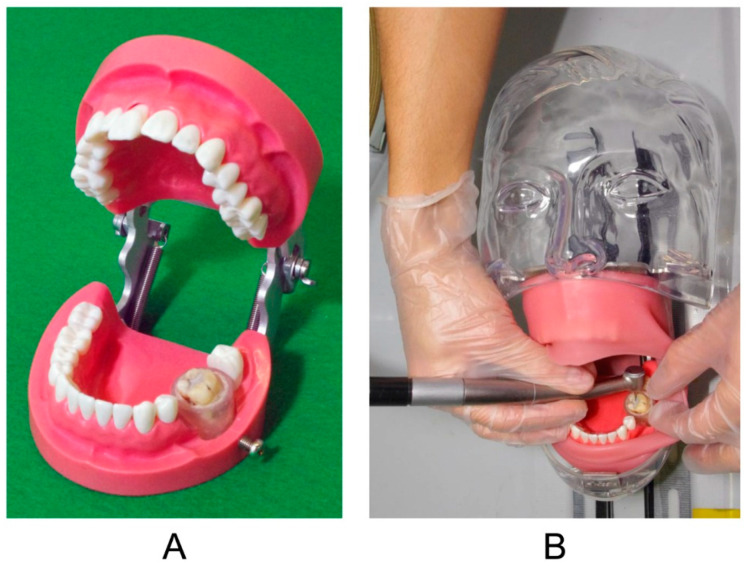
Tooth setting in the phantom head. (**A**) Prepared tooth placed on the left first molar position, (**B**) entire image of the phantom head with the prepared tooth.

The prepared tooth was set to the left first molar position of a phantom head (Simple Manikin III, Nisshin, Tokyo, Japan) with a rubber cover (SPM III, Nisshin, Tokyo, Japan) mimicking the buccal mucosa (Figure 2). An air turbine (twin-power turbine, Morita, Osaka, Japan) with a carbide bur (FG 699, Shofu, Kyoto, Japan) was used to remove dental amalgams. The rubber cover and carbide burr were changed to new items for every dental amalgam removal.

### 2.3. Removal of the Dental Amalgam

Removal of dental amalgam from the tooth was performed using two methods: block system (BLK) and fragment system (FRG). For the BLK, the tooth structure surrounding the dental amalgam was cut to avoid contact between the bur and the dental amalgam, and then removed as a single block using an excavator (Round Excavator S-0.7, GC, Tokyo, Japan). For the FRG, dental amalgam was removed as fragments by performing cross-shape cutting, but not touching the tooth structure with the bur. In addition, the experiments removing dental amalgam were carried out with two factors (four conditions): with or without cooling water (WW or NW) and with or without an intraoral suction (WI or NI). Dental amalgam removal was performed by a single experienced dentist in a fume hood.

### 2.4. Collecting the Samples and Measurement of Mercury Amount

**Figure 3:**
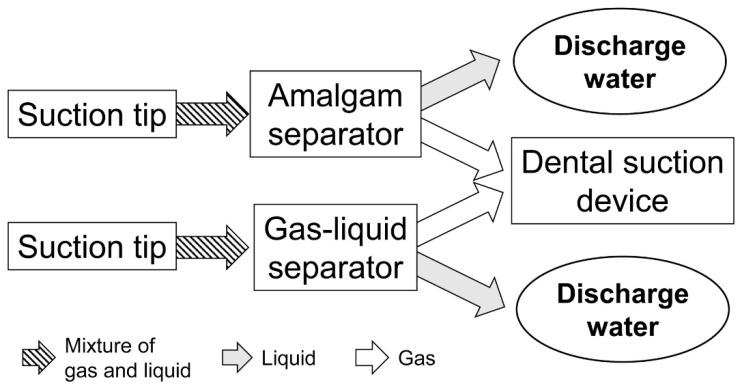
Schematic of the drainage system.

After removing the dental amalgam, the oral cavity of the phantom head was washed using 100 mL of deionized water to mimic the patient’s mouse rinse, and the water was collected as samples of “oral rinse water”. Furthermore, to clean the inside of the drainage system, 500 mL of deionized water was suctioned and collected as samples of “system rinse water”. A schematic of the drainage system is shown in Figure 3. For both the oral rinse water and system rinse water, samples were collected by two methods: drained water captured through an amalgam separator (CAS 1, Dürr Dental, Bietigheim-Bissingen, Germany) as method “AM”, and through a gas–liquid separator (7 L separator, Tokyo Giken, Tokyo, Japan, capacity: 7.0 L) as method “GL”. A suction dental device (TCV-CS1000, Tokyo Giken, Tokyo, Japan; suction power: 17.65 kPa) was connected in both methods. For measuring the mercury amount, a highly-sensitive mercury analyzer (WA-5A, Nippon Instruments, Tokyo, Japan), which used vapor deposition of mercury with gold foil and atomic absorption spectrometry, was used. Measurements were performed on the same day of sample collection.

### 2.5. Statistics Analysis

The number of repetitions was set to five (*n* = 5). Regarding the results of the mercury amount in the oral rinse water and system rinse water, four-way analysis of variance (ANOVA) was performed (factor A: removal methods (BLK and FRG), factor B: the absence of cooling water (WW and NW), factor C: the absence of intraoral suction (WI and NI), and factor D: drainage systems (AM and GL)). Tukey’s multiple comparisons were performed when significant differences were observed in the factors and their interactions (α = 0.05).

## 3. Results

The mean values and standard deviations of mercury amount in each condition are shown in Table 1. In the oral rinse water, the greatest value showed in the conditions of FRG, NW, WI, and GL (39,525 μg/m^3^), and the smallest value in the condition of FRG, NW, NI, and AM (5204 μg/m^3^). In the system rinse water, the greatest value showed in the conditions of BLK, NW, WI, and AM (20,802 μg/m^3^), and the smallest value in the condition of BLK, WW, WI, and GL (4031 μg/m^3^). Both the greatest and smallest values of the system rinse water were smaller than those of oral rinse water.

**Figure 4:**
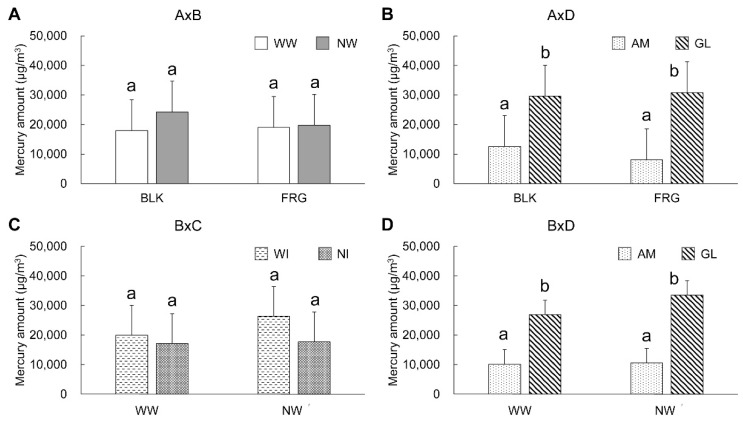
Mercury amounts in the oral rinse water. Error bar indicates standard deviation. Same letter designation indicates nonsignificant difference (*p* > 0.05). (**A**) Interaction of removal methods (block system: BLK, fragment system: FRG) with absence of cooling water (with cooling water: WW, no cooling water: NW), (**B**) Interaction of removal methods with difference of drainage system (amalgam separator: AM, gas-liquid separator: GL), (**C**) Interaction of absence of cooling water with absence of intraoral suction (with intraoral suction: WI, no intraoral suction: NI), (**D**) Interaction of absence of cooling water with difference of drainage system.

Regarding the results of the oral rinse water, a significant difference (*p* < 0.05) was found in factor A, and highly significant differences (*p* < 0.01) in B, C, and D, and the interactions A × B, A × D, B × C, and B × D. According to the results of Tukey’s multiple comparisons, a significantly smaller mercury amount was found in NI (17,384 μg/m^3^) and AM (10,316 μg/m^3^) than in WI (23,114 μg/m^3^) and GL (30,182 μg/m^3^), respectively (*p* < 0.01). However, there were no significant differences between BLK (21,083 μg/m^3^) and FRG (19,415 μg/m^3^) as well as between WW (18,499 μg/m^3^) and NW (21,999 μg/m^3^) (*p* > 0.05) (Table 2). Figure 4 shows the graphs of the two-factor interactions that were significantly different according to the four-way ANOVA (*p* < 0.01). In the interaction of A × B (Figure 4A), in BLK, a higher mercury concentration seemed to be found in NW than in WW, but there was no significant difference between them (*p* > 0.05). On the other hand, in the FRG, the results showed almost the same values between NW and WW. In the interaction of A × D (Figure 4B), GL showed a significantly higher mercury amount than AM in both BLK and FRG (*p* < 0.01), but there were no significant differences between the removal methods in each drainage system (*p* > 0.05). In the interaction of B × C (Figure 4C), WI showed a higher mercury amount than NI in both WW and NW conditions, but no significant difference was found between them (*p* > 0.05). In the interaction of B × D (Figure 4D), GL showed a significantly higher mercury amount than AM in both WW and NW (*p* < 0.01). There was no significant difference between WW and NW in each drainage system (*p* > 0.05).

**Figure 5:**
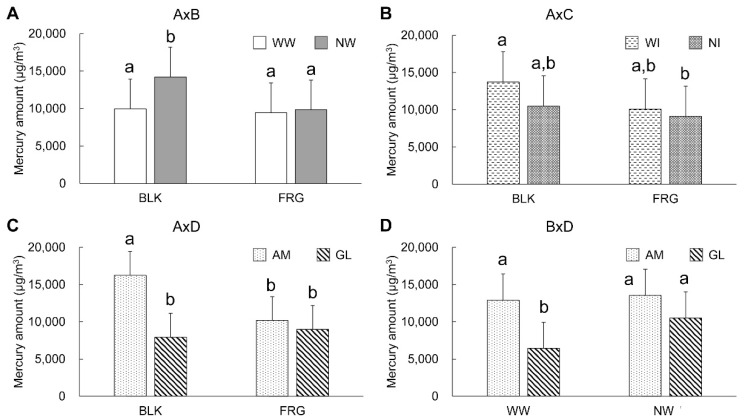
Mercury amounts in the system rinse water. Error bar indicates standard deviation. Same letter designation indicates nonsignificant difference (*p* > 0.05). (**A**) Interaction of removal methods (block system: BLK, fragment system: FRG) with absence of cooling water (with cooling water: WW, no cooling water: NW), (**B**) Interaction of removal methods with absence of intraoral suction (with intraoral suction: WI, no intraoral suction: NI), (**C**) Interaction of removal methods with difference of drainage system (amalgam separator: AM, gas-liquid separator: GL), (**D**) Interaction of absence of cooling water with absence of intraoral suction.

Regarding the results of the system rinse water, a significant difference (*p* < 0.05) was found in the interaction A × C, and highly significant differences (*p* < 0.01) in all factors (A, B, C, and D), as well as the interactions A × B, A × D, and B × C. According to the results of Tukey’s multiple comparisons, significantly smaller mercury was found in FRG (9598 μg/m^3^), WW (9658 μg/m^3^), NI (9796 μg/m^3^), and GL (8467 μg/m^3^) than BLK (12,093 μg/m^3^), NW (12,034 μg/m^3^), WI (11,896 μg/m^3^), and AM (13,224 μg/m^3^), respectively (*p* < 0.05). Figure 5 shows the graphs of the two-factor interactions, which showed significant differences according to four-way ANOVA. In the interaction of A × B (Figure 5A), WW showed a significantly higher mercury amount than MW in BLK (*p* < 0.01), but there was no significant difference between WW and NW in FRG (*p* > 0.05). NW in BLK showed a significantly higher amount of mercury than both WW and NW in FRG (*p* < 0.01) (Table 3). In the interaction of A × C (Figure 5B), WI in BLK showed a significantly higher mercury amount than NI in FRG (*p* < 0.01), but there were no significant differences among other combinations (*p* > 0.05). In the interaction of A × D (Figure 5C), AM in BLK was significantly higher than in other conditions (*p* < 0.01), but there were no significant differences among other combinations (*p* > 0.05). The interaction of A × B (Figure 5D) was significantly smaller for WW in GL than for other conditions (*p* < 0.01).

## 4. Discussion

The amounts of mercury remaining in the oral cavity and inside the drain system after dental amalgam removal under various conditions were measured as “oral rinse water” and “system rinse water”, respectively. Consequently, the removal conditions affected the results in both the oral rinse water and the system rinse water.

Dental amalgam cannot be used for esthetic restorations because of its metallic color. White materials, such as composite resins, are more appropriate for restorations, and it is easy to predict an increase in the use of white materials to meet the demand for esthetic restorations [27]. However, dental amalgam has been used for many years; therefore, there will be many chances to remove them from the patient’s mouth [28]. In general, the removal of restoration from the tooth is performed using a high-speed rotary cutting apparatus. However, this procedure generates heat in the restoration materials due to friction between the cutting instruments and materials. In the case of dental amalgam removal, the filling is also heated during the procedure and releases mercury into the environment [28,31]. Once mercury is released into the environment, marine organisms cause its bioconcentration, and our health might be harmed by the consumption of the fish [24,27]. Therefore, it is especially important to reduce the amount of mercury in the discharged water. The drainpipe would be contaminated with mercury as it drains the discharged water that contains mercury [16,29]. Thus, it is important that to clarify the mercury amount left in the system after the procedure to prevent mercury pollution of the environment.

In this study, dental amalgam was removed using two different methods. In a safe protocol for dental amalgam removal published in 2012, dental amalgam was cut using drilling instruments [14]. However, drilling dental amalgams increases mercury vapor because the drilling device, such as air turbines, spins the bur or point at 350,000 rpm and generates friction heat [28,31]. To minimize the effect of the mercury vapor produced by friction heat during dental amalgam removal, we also investigated a non-clinical removal method of “block system”, in which the tooth structure surrounding the dental amalgam was cut, not touching the bur to the dental amalgam. The amount of mercury discharged from amalgam removal was measured under various conditions: removal methods, absence of cooling water and intraoral suction, and drainage systems. In clinical practice, water spray and suction are commonly used during teeth drilling to protect their pulp from the heat explained above; therefore, there is little chance of drilling teeth without using water spray and suction. However, in non-clinical situations, dental students report removing dental amalgams without water spray and suction to ensure visibility during their training [31]. Thus, it was essential to investigate the effects of the absence of cooling water and intraoral suction on the amount of mercury.

In the oral rinse water, the results showed how mercury was left after amalgam removal in the oral cavity. There was no significant difference between the removal methods and the absence of cooling water. The cutting instrument did not directly touch the dental amalgam fillings during the BLK removal procedure. However, heat might be produced due to friction between the tooth structure and bur, releasing mercury from the dental amalgam fillings. In contrast, using the intraoral suction and amalgam separators showed significantly lower mercury amounts. These results show that intraoral suction could prevent the scattering of amalgam particles into the oral cavity during the removal procedure. Some studies have investigated mercury vapor while removing dental amalgam fillings [30,31,32]. They reported that using a cooling spray and intraoral suction could reduce mercury levels during the procedure. However, there was no significant difference in the absence of cooling water in the present study. This means that cooling water can reduce mercury during removal of dental amalgams, but it is challenging to reduce the mercury remaining in the oral cavity after the procedure because mercury produced during the procedure might be suctioned or coming out from the oral cavity. The amalgam separator captured amalgam particles above a certain size [16]. Consequently, the mercury amount of the amalgam separator was significantly smaller than that of the gas-liquid separators. In clinical situations, rubber dam application is essential for removing dental amalgam restorations [14]. However, for simplification of the experiments, we did not use rubber dams.

In the system rinse water, the results showed how mercury was left after the removal of amalgam in the drain. Removing the fragment system and using water spray and intraoral suction resulted in significantly lower mercury amounts. However, the amalgam separator did not significantly reduce the mercury amount. The amalgam separator cannot catch amalgam particles smaller than a certain size, but it is said that the cutting instrument creates very fine particles in the amalgam removal procedure [16]. Owing to their size and weight, the large particles would be drained during the removal procedure and could not remain in the drainpipes or hoses. In addition, it is impossible to collect dissolved mercury from the discharged water [16]. Consequently, the amalgam separator cannot sufficiently reduce mercury, and it is suggested that collecting mercury completely from the discharge water is challenging even with the amalgam separator. However, there are some reports that amalgam separators can significantly reduce mercury from discharged water with large amounts of mercury; therefore, the use of separators is required for dental offices by law in some countries [16,27]. Thus, the use of amalgam separators is recommended to drain discharged water during and after removing dental amalgams.

The limitation of this study is that this experiment was ex vivo setting. Besides, it was difficult to use rubber dams in this study because the undercut of teeth for putting them was missing due to the self-curing resin. The use of rubber dams helps to reduce the mercury from the oral cavity so that the amount might decrease compared to our results. The non-clinical removal method, the block system, did not help to reduce the amount of mercury in both the oral and system rinse water. On the contrary, this method removes the sound tooth structure. That is against minimal intervention dentistry, so a fragment system would be a better way to remove dental amalgam. In Japan, comprehensive legislation and regulatory frameworks for handling mercury waste have been developed. According to the guidelines, the discharge water must not contain more than 5000 µg/m^3^ of mercury [37]. However, the amount of mercury obtained in this study was higher than this value. In fact, an extremely high amount of mercury has been drained from dental offices than in this study [16]. The condition in this study was slightly different from the actual condition, such as the total amount of water discharged and the components. However, the reduction of releasing mercury into the environment is mandatory, and our results would be evidence to help to save from mercury pollution. In addition, mercury produced during amalgam removal might have been released from the oral cavity as an aerosol [28,30,31,32]. Therefore, further studies are needed to clarify their mercury levels. From the viewpoint of environmental and health protection, we must reduce the use of amalgams containing high levels of mercury and make an effort not to drain mercury to the environment by using proper methods to remove amalgam restorations and using amalgam separators.

## 5. Conclusions

It was clarified that mercury in the oral cavity and drainage system after removing dental amalgams could be reduced when amalgams are removed by being cut into fragments and using cooling water and intraoral suction. In addition, using amalgam separators can significantly reduce mercury from the discharge water and prevent the draining of mercury into the environment.

## Figures and Tables

**Table 1:** The mean values and standard deviations of mercury amount in each condition.

(Factor A)	(Factor B)	(Factor C)	(Factor D)	Oral Rinse Water		System Rinse Water	
Removal Method	Cooling Water	Intraoral Suction	Drainage Systems	Mean (*S.D.*) [μg/m^3^]		Mean (*S.D.*) [μg/m^3^]	
Block system (BLK)	With (WW)	With (WI)	Amalgam separator (AM)	13,203	(1990)	17,251	(1136)
			Gas–liquid separator (GL)	25,416	(2212)	4031	(611)
		Without (NI)	Amalgam separator (AM)	10,389	(2951)	14,195	(6078)
			Gas–liquid separator (GL)	22,835	(10,589)	4358	(2632)
	Without (NW)	With (WI)	Amalgam separator (AM)	17,779	(1937)	20,802	(2054)
			Gas–liquid separator (GL)	37,784	(1785)	12,757	(1930)
		Without (NI)	Amalgam separator (AM)	8901	(1482)	12,764	(3727)
			Gas–liquid separator (GL)	32,359	(6351)	10,589	(4172)
Fragment system (FRG)	With (WW)	With (WI)	Amalgam separator (AM)	9525	(2050)	11,825	(1707)
			Gas–liquid separator (GL)	31,440	(955)	8328	(1162)
		Without (NI)	Amalgam separator (AM)	7280	(868)	8344	(648)
			Gas–liquid separator (GL)	27,902	(2777)	8930	(324)
	Without (NW)	With (WI)	Amalgam separator (AM)	10,242	(1042)	8928	(400)
			Gas–liquid separator (GL)	39,525	(1800)	11,242	(755)
		Without (NI)	Amalgam separator (AM)	5204	(426)	11,683	(1273)
			Gas–liquid separator (GL)	24,201	(1999)	7504	(860)

**Table 2:** The mean values and standard deviations of mercury amount of four-way ANOVA’s main factors in the oral rinse water.

Factors		Conditions	Mean (*S.D.*) [μg/m^3^]		
A	Removal method	Block system (BLK)	21,083	(10,648)	NS
		Fragment system (FRG)	19,415	(12,210)	
B	Cooling water	With (WW)	18,499	(9600)	NS
		Without (NW)	21,999	(12,867)	
C	Intraoral suction	With (WI)	23,114	(11,491)	**
		Without (NI)	17,384	(10,742)	
D	Drainage systems	Amalgam separator (AM)	10,316	(3903)	**
		Gas–liquid separator (GL)	30,182	(7161)	

NS indicates no significant difference (*p* > 0.05) and ** for significant differences (*p* < 0.01) between the conditions in each factor.

**Table 3:** The mean values and standard deviations of mercury amount of four-way ANOVA’s main factors in the system rinse water.

Factors		Conditions	Mean (*S.D.*) [μg/m^3^]		
A	Removal method	Block system (BLK)	12,093	(6151)	**
		Fragment system (FRG)	9598	(1828)	
B	Cooling water	With (WW)	9658	(4834)	**
		Without (NW)	12,034	(4254)	
C	Intraoral suction	With (WI)	11,896	(5052)	**
		Without (NI)	9796	(4069)	
D	Drainage systems	Amalgam separator (AM)	13,224	(4617)	**
		Gas–liquid separator (GL)	8467	(3415)	

** indicates significant differences (*p* < 0.01) between the conditions in each factor.
